# Cellular Responses to Flavivirus Infections: Stress Signaling at the Crossroads of Host Defense and Virus Infection

**DOI:** 10.3390/v18070748

**Published:** 2026-07-07

**Authors:** Pheonah Badu, Elianna T. Cruz González, Cara T. Pager

**Affiliations:** 1 Department of Biological Sciences, University at Albany, State University of New York, Albany, NY 12222, USA; 2 The RNA Institute, University at Albany, State University of New York, Albany, NY 12222, USA

**Keywords:** flaviviruses, unfolded protein response, integrated stress response, immune response, apoptosis, autophagy

## Abstract

Flaviviruses, encompassing notable pathogens, like Dengue, Zika, West Nile, and tick-borne encephalitis viruses, elicit complex cellular stress responses, involving pathways such as the unfolded protein response (UPR), integrated stress response (ISR), apoptosis, autophagy, and the antiviral immune response. These pathways regulate cell fate by either promoting survival to counteract virus-induced damage or triggering cell death programs under prolonged and irreparable stress. Therefore, the primary aim of flavivirus-induced cellular responses is to protect cells and hinder viral propagation. Despite cellular defenses, flaviviruses have evolved various subversion strategies, mainly involving viral proteins, which enable successful infections even when cellular responses are activated. While these cellular pathways were previously perceived as separate entities, recent studies suggest interplay and dynamic shifts among these stress response pathways, underscoring the need for further investigation in this area. In this review, we explore the key pathways activated during flavivirus infections, examine mechanisms of viral subversion, and delve into the synergy of these pathways, thereby elucidating the impact on the progression of infection. A deeper understanding of these interactions will guide future efforts to define how cellular stress responses shape flavivirus infection and leverage this knowledge toward the development of targeted antiviral strategies.

## 1. Introduction

Many viruses within the genus *Orthoflavivirus* (hereafter referred to as flavivirus) are significant human pathogens due to their capacity for emergence and re-emergence [1]. Within the *Flaviviridae* family, the flavivirus genus comprises more than 70 defined members [2]. Key members of the group include Dengue virus (DENV), yellow fever virus (YFV), West Nile virus (WNV), Japanese encephalitis virus (JEV), Zika virus (ZIKV) and tick-borne encephalitis virus (TBEV). These viruses are primarily transmitted to humans by arthropod vectors (such as mosquitoes and ticks) and have a substantial toll on human health, with DENV alone accounting for ~390 million infections each year [1,3]. Flavivirus infections are often associated with mild symptoms but can result in severe outcomes such as encephalitis, hemorrhagic disease, and neuronal complications as in the case of JEV, DENV and ZIKV infections, respectively [4]. With the surge in disease severity, climate change influencing the global expansion of vectors, lack of licensed antiviral drugs and limited vaccines available for some members of this genus, flavivirus infections remain a significant public health concern [4,5,6].

Flaviviruses are enveloped cytoplasmic viruses with a plus-sense single-stranded RNA genome approximately 11 kilobases in length [2]. The genome is non-segmented and contains a coding region bordered at the 5′- and 3′-ends by untranslated regions (UTRs). The coding sequence encodes structural proteins (capsid [C], pre-membrane/membrane [prM/M], envelope [E]) that make up the viral particle and nonstructural proteins (NS1, NS2A, NS2B, NS3, NS4A, NS4B, NS5) essential for viral replication, packaging, and impairing the host immune response [2,7]. Besides these features, the flavivirus genome possesses a 5′-methyl guanosine cap but no poly(A) tract at the 3′-terminus of the viral RNA [8,9]. Using the host cellular translation machinery and the membrane of the endoplasmic reticulum (ER), a single multi-pass transmembrane viral polyprotein is produced [9]. Both viral and cellular proteases process the polyprotein into individual viral proteins, and the nonstructural proteins support the assembly of the replication factories within the ER membrane [9,10]. Within these factories the viral RNA serves as a template to make more copies of the viral RNA genome via a negative-sense RNA intermediate [11,12,13,14]. After new viral RNA genomes are synthesized, the RNA is transferred to assembly factories on the ER where the viral RNA is enclosed within viral protein shells to form new virions. The ER therefore provides a critical platform that supports the various aspects of the flaviviral life cycle [14], undergoing significant enlargement and remodeling to accommodate these processes [12]. Beyond the ER, flavivirus infection triggers elaborate cytoskeletal rearrangements and disruption of cell signaling pathways [15]. While these intrinsic changes favor the virus, normal cellular organization and processes are also perturbed and induce various types of cellular stress, which rewires the transcriptional program to mitigate the resulting stress. The major signaling pathways activated to combat the ensuing stress and obstruct viral events include the unfolded protein response (UPR), the integrated stress response (ISR), and antiviral immune response. The main components of such flavivirus-induced host responses, the consequence of the host response activation on flaviviruses, potential crosstalk between these pathways and the possible subversion strategies employed by flaviviruses against these responses are the focus of this review.

## 2. Cellular Stress Responses

Flavivirus infection activates multiple host stress pathways, including the unfolded protein response (UPR), the integrated stress response (ISR), and innate immune signaling. These responses act in concert to limit infection and promote host survival, although flaviviruses can also subvert stress signaling to support replication. The following sections examine how these pathways are engaged and regulated during flavivirus infection.

### 2.1. The Unfolded Protein Response (UPR) Pathway

**Figure 1:**
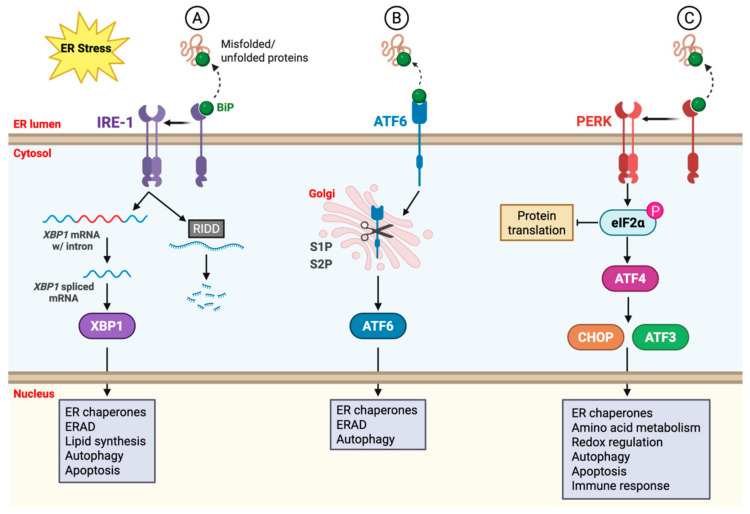
The unfolded protein response (UPR) pathway. Flavivirus replication on and rearrangement of the ER membrane induces ER stress and stimulates the UPR. The stress is mitigated by signaling through three key transactivating receptors: (**A**) IRE-1, (**B**) ATF6 and (**C**) PERK. Activation of these receptors alleviates ER stress by repressing anti-apoptotic genes or initiating a pro-apoptotic program, ultimately leading to apoptosis. Alternatively, the UPR can prime the cells to mount a robust antiviral response, serving as a defense mechanism against viral infection [16,17,18,19,20]. ERAD: ER-associated degradation; RIDD: regulated IRE-1-dependent decay; S1P and S2P: Golgi proteins site-1 and -2 proteases. Figure was adapted from [16]. Created in BioRender. Pager, C. (2026) https://BioRender.com/thq6e28 (accessed on 30 June 2026).

The UPR is an ER stress program activated by different signal transduction pathways [16]. Sensors embedded in the ER membrane, including inositol-requiring enzyme 1 α and β (IRE-1α and IRE-1β), activating transcription factor 6 (ATF6) and PKR-like ER kinase (PERK), initiate the UPR and downstream effector proteins that induce transcriptional programs to alleviate stress or activate cell death (Figure 1) [16]. In a healthy cellular environment, the ER chaperone BiP (also known as GRP-78) interacts with the ER luminal domains of the UPR sensors and renders these receptors inactive. However, in response to the stress induced by the accumulations of misfolded and unfolded proteins in the ER lumen, BiP uncouples from the UPR receptors to aid protein refolding within the ER lumen (Figure 1). This disengagement of BiP from the sensors activates the receptors to initiate the UPR and receptor-specific downstream signaling pathways [17].

The IRE-1 receptor is a type I transmembrane protein that has ribonuclease and kinase domains within the cytoplasmic region (Figure 1A) [16]. Upon ER stress conditions such as the accumulation of misfolded or unfolded proteins, BiP dissociates from the IRE-1 receptor to aid protein refolding. This disengagement of BiP from the IRE-1 receptor initiates receptor dimerization, trans-autophosphorylation and IRE-1 activation [16]. Upon IRE-1 activation, the ribonuclease domain within the IRE-1 cytoplasmic tail excises a 26-nucleotide intron from *XBP1* to generate a spliced mature *XBP1* mRNA. Subsequent translation of the pro-cell survival transcriptional activator XBP1 promotes transcription of cellular transcripts involved in protein folding and quality control including ER chaperone activities, autophagy, ER-associated protein degradation (ERAD) and lipid biosynthesis critical to ER expansion (Figure 1) [18,19]. IRE-1 also participates in another process known as regulated IRE-1-dependent decay (RIDD), which entails the degradation of mRNAs localized to the ER [20,21,22]. ER-localized mRNAs targeted for RIDD have cleavage sites like those in *XBP1* that are recognized by the IRE-1 ribonuclease. The resulting mRNA fragments are subsequently degraded by cellular exoribonucleases [20]. IRE-1 exists in two isoforms, IRE-1α and IRE-1β, and both can splice *XBP1* and activate RIDD; however, IRE-1α exhibits stronger splicing activity, while IRE-1β provokes a stronger RIDD function [23]. In addition to the functions related to XBP1 or the promotion of the RIDD pathway, IRE-1α also associates with TNF receptor-associated factor 2 (TRAF2). This interaction results in the recruitment of apoptosis signal-regulating kinase 1 (ASK1), which then interacts with TRAF2 [24]. Once activated, ASK1 initiates signaling cascades involving c-Jun amino-terminal kinase (JNK) and p38 that subsequently initiate the apoptotic pathway [25].

ATF6 is another transmembrane UPR sensor that belongs to the basic leucine zipper (bZIP) family of transcription factors (Figure 1B) [26]. The type II transmembrane ATF6 protein contains a transcription and activation domain within the cytoplasmic domain and a Golgi localization signal sequence in the ER luminal region. ATF6 remains inactive until the dissociation of BiP, which exposes the Golgi localization motif leading to the trafficking of ATF6 to the Golgi where the protein is proteolytically cleaved by the resident Golgi proteins site-1 and -2 proteases (S1P, S2P) [17,27]. The released cytosolic domain of ATF6 is imported into the nucleus and activates transcription of downstream target genes involved with ER chaperone expression, ERAD, and autophagy to regulate stress (Figure 1B) [27,28].

**Figure 2:**
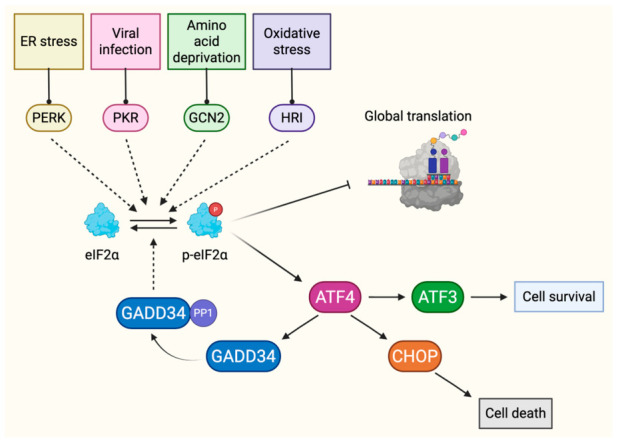
The integrated stress response (ISR) pathway. Activation of four kinases (PERK, PKR, GCN2 and HRI) in response to specific stressors such as ER stress, viral infection, nutrient deprivation, and oxidative stress initiates the ISR. Signaling from these kinases leads to phosphorylation of eIF2α, which inhibits global protein synthesis and at the same time selectively promotes translation of specific stress response mRNAs such as *ATF4*. ATF4 supports transcriptional programs to restore cellular homeostasis, promote cell survival, or trigger apoptosis. ATF4 also directs the expression of GADD34, which, when partnered with PP1, dephosphorylates eIF2α to restore global translation. GADD34: growth arrest and DNA damage-inducible protein 34; PP1: protein phosphatase 1. Figure adapted from [82,83]. Created in BioRender. Pager, C. (2026) https://BioRender.com/reo01s6 (accessed on 30 June 2026).

PKR-like endoplasmic reticulum kinase (PERK) is a member of the eIF2α kinase subfamily and the third UPR sensor (Figure 1C) [29]. The structure of PERK resembles that of IRE-1, having a type I transmembrane protein architecture comprising both luminal BiP binding and cytoplasmic kinase domains. Like IRE-1, the binding of BiP to the luminal domain of PERK keeps the receptor inactive [29]. As unfolded and misfolded proteins accumulate in the ER, BiP dissociates from PERK due to its higher affinity for these proteins. Consequently, PERK monomers oligomerize and undergo trans-autophosphorylation within the kinase domain, enabling full catalytic activity and the initiation of the UPR and ISR signaling cascade (Figure 1C and Figure 2) [29]. Activated PERK directly phosphorylates of eIF2α on serine 51, leading to the suppression of global translation initiation. This inhibition of translation initiation promotes the selective noncanonical modes of protein synthesis such as the bypassing of the inhibitory upstream open reading frame (uORF), thereby enabling the translation of specific mRNAs such as Activating Transcription Factor 4 (*ATF4*), which orchestrates the stress response program [30]. Once translocated to the nucleus, ATF4 directs the transcription of various genes, e.g., *ATF3* and *CHOP* (also known as *DDIT3*) [31,32,33]. These genes regulate cellular adaptive responses that help mitigate stress, such as autophagy, redox regulation, apoptotic signaling, and the immune response (Figure 1) [31,32,33].

### 2.2. Flaviviruses Interface with the UPR During Infection

The UPR is pivotal in restoring proper protein folding, preserving ER homeostasis, and synergizing with antiviral mechanisms to fight viral invasions. Flaviviruses, however, usurp the UPR pathways to promote virus replication within host cells [34]. The mechanisms of this manipulation, though not completely understood across all flaviviruses, involve intricate interactions between viral components and UPR signaling molecules and lead to differential activation of IRE-1, ATF6 and PERK pathways [34,35,36,37,38,39,40,41].

#### 2.2.1. Flavivirus Proteins and Regulation of the UPR

Flavivirus proteins, notably the nonstructural proteins, are instrumental in modulating UPR pathways mainly by directly engaging with UPR factors [40]. Many studies have highlighted the explicit role of flaviviral proteins, particularly those that localize to the ER, in regulating UPR signaling to limit host defenses. In the case of WNV, nonstructural proteins NS1-NS5 have been shown to initiate the UPR, causing expression of CHOP, a proapoptotic factor that acts to restrict WNV infection [39]. Similarly, the WNV Kunjin strain selectively activates the ATF6 and IRE-1 pathways to enhance replication and potentially suppress antiviral response, with NS4A and NS4B proteins as the key activators of the response [38]. DENV prM, E, and NS1 glycoproteins and the smaller hydrophobic proteins like NS2A, NS2B-NS3, and NS4B have been shown to robustly activate the IRE-1 pathway. As a result, cell survival is enhanced, and the timeframe for viral replication is extended [36]. Moreover, interactions between DENV E protein and ER chaperones like calnexin, calreticulin, and BiP were demonstrated through co-immunoprecipitation and co-localization experiments. These interactions were associated with increased virus production likely promoting the correct folding of the viral glycoprotein, facilitating viral protein assembly and preserving proteostasis in the ER [42]. Similarly, JEV co-opts the ER chaperone BiP to support virion assembly and particle formation [43]. While BiP was not required for viral RNA replication, this ER chaperone was critical for the production of fully mature and infectious particles, thereby enhancing viral fitness and the efficiency of subsequent infections [43]. Overall, flavivirus proteins act as key regulators of UPR sensors, coordinating ER stress responses to favor viral persistence.

#### 2.2.2. The Role of the UPR in Flavivirus Infection

The UPR is central to host adaptation and defense, enhancing protein folding and processing, membrane biogenesis and regulating cell survival [44]. Interestingly, UPR-associated processes can be manipulated by flaviviruses to promote viral infection (Table 1) [38]. Mosquito-borne flaviviruses, such as DENV, WNV, ZIKV and JEV, as well as tick-borne flaviviruses like Langat virus (LGTV), activate the three branches of the UPR, albeit differentially and in a temporal manner [36,38,45]. For instance, DENV infection triggers an initial activation of PERK, followed by the sequential activation of ATF6 and IRE-1 during the mid and late stages of infection, respectively [35,37,46]. This stepwise activation of the UPR branches enables the virus to circumvent the restriction on protein synthesis to promote the production of viral proteins required for virion assembly [35]. Further research demonstrates that DENV activates the IRE-1 pathway, evidenced by the splicing of *XBP1* mRNA, and the elevated expression of downstream targets, *ERdj4*, *EDEM1* and *p58* [36]. By triggering the XBP1 signaling pathway, DENV mitigates virus-induced cytotoxicity in the ER and promotes viral survival [38]. Interestingly, DENV infection in mouse embryonic fibroblast (MEF) cells deficient in IRE-1, PERK and ATF6 showed differential infection outcomes [35]. Less infectious DENV was produced in IRE-1-/- MEFs compared to wild-type cells, while higher amounts of DENV were released in the PERK-/- MEFs. Notably, knockout of ATF6 had no effect on DENV infection kinetics or virus titers [35]. As expected, when the UPR was stimulated prior to infection, DENV infection was markedly suppressed due to the preemptive signaling cascade, which also primed the innate immune response [46]. During JEV infection, Protein mono-ADP Ribosyltransferase (PARP-16), a protein known to inhibit translation and regulate the UPR, was shown to control the activation of PERK and IRE-1 in neural stem cell progenitor cells [47]. Notably, JEV infection induced IRE-1 signaling, and this positively affected JEV replication and further enhanced chaperone expression [36]. This enhancement of chaperone expression by DENV and JEV via the strategic activation of ER stress response pathways, highlights the sophisticated mechanisms flaviviruses employ to ensure their proliferation and survival within host cells.

In a mouse infection model, ZIKV was also shown to activate the IRE-1 pathway and XBP1 transcriptional programming, which enhanced virus replication [48]. In A549 cells, ZIKV infection induced the UPR by activating PERK and *XBP1* splicing early in infection at 16 h post-infection, followed by sustained activation of PERK, IRE-1 and ATF6 receptors during the later stages of infection [41]. The prolonged activation culminated in a shutdown of protein synthesis, cell lysis and viral release at the later stage of infection [41]. Furthermore, ZIKV significantly diminished the transcription and translation of BiP, thus dampening the BiP response and sustaining the activation of UPR sensors, which overall facilitated viral replication [43]. Notably, however, the modulation of the UPR leading to inhibition or hyperactivation of IRE-1 endonuclease function affected ZIKV titers and the immune response in human microglial cells, where IRE-1 and XBP1 also localized to replication sites [49]. In the broader context of infection, the UPR induced by ZIKV infection contributes to ZIKV-associated microcephaly [50]. Studies examining ZIKV infection of human fetuses, cultured human neural stem cells and mouse embryos showed that ZIKV induced ER stress and UPR signaling in vivo and in vitro. Consequently, infected neural projector cells produced fewer projections and the sustained UPR led to apoptosis, which contributed to the microcephaly phenotype [50]. Consistent with these findings, Tan and colleagues, in an independent study of ZIKV-infected mouse brain and human neural cells, illustrated robust ATF6 and IRE-1 signaling. This induction similarly led to the upregulation of downstream targets CHOP and BiP in vivo and in vitro, potentially contributing to the neuropathology associated with ZIKV infection [51]. In contrast, ZIKV infection in A549 cells delayed the onset of apoptosis by hindering the expression of CHOP protein [52].

WNV infections in neuronal cells equally highlighted the importance of the UPR for viral replication and showed distinct effects of the virus on each of three branches of the UPR. This led to chaperone activation, translational repression, and *CHOP* transcription [39]. Remarkably, despite activation of IRE-1, this pathway was not crucial for WNV replication as levels of WNV gene expression remained similar in both *XBP1*-depleted and control cells [39]. In contrast, activation of PERK played a host-protective role, leading to a decrease in WNV titers [39]. Curiously, studies with the WNV Kunjin strain similarly demonstrated activation of the UPR; however, in these infections, the virus induced IRE-1 and ATF6 signaling. This strategic regulation of the UPR supported viral replication by minimizing PERK activation, which otherwise would limit synthesis of viral proteins [38]. Additionally, it was reported that WNV Kunjin manipulation of PERK signaling was connected with ATF6 activation as cells lacking ATF6 had enhanced PERK signaling, which coincided with diminished virion production [53]. Infection with Usutu virus (USUV), a close relative of WNV and JEV, also triggered the IRE-1 arm of the UPR, as evidenced by an increase in *XBP1* splicing following infection [54].

In TBEV, genome-wide analysis showed an upregulation of UPR-related genes, including *XBP1* and *CHOP*, early in infected U2OS cells [55]. Infection with two different TBEV strains, Neudoerfl and MucAr-H-171/11, was also shown to trigger the IRE-1 pathway. Notably, the neurotropic Neudoerfl strain strongly activated XBP1 in astrocytes, neuronal and intestinal cells, while MucAr-HB-171/11 activated the pathway in intestinal and neuronal cells but not astrocytes [56]. The resulting IRE-1 activation significantly enhanced viral replication, which was diminished upon inhibition of the pathway [56]. The related but less pathogenic Langat virus (LGTV) similarly induced IRE-1 albeit to a lesser extent in astrocytes and intestinal cells [56]. Interestingly, while LGTV appeared to counteract ATF6 and IRE-1 pathways, the virus is restricted by the antiviral effects mediated by PERK [57].

Consistent with observations in other flaviviruses, the avian-derived Tembusu virus (TMUV) triggered ER stress, leading to the activation of the UPR [58]. Recent findings indicated that TMUV infection prompted a significant increase in ER chaperones, specifically BiP and glucose-regulated protein 94 (GRP94). At the onset of infection, the PERK pathway was activated following infection and resulted in elevated levels of *ATF4*, *GADD34*, and *CHOP*. Concurrently, the IRE-1 pathway was engaged, leading to the splicing of *XBP1* mRNA and enhanced p58IPK expression, a downstream target of spliced *XBP1* that negatively regulates stress-induced eIF2α signaling [58,59]. Additionally, TMUV increased ATF6 expression and signaling, which correlated with the upregulation of downstream chaperones such as calnexin, calreticulin, ERp57, and PDI [58].

**Table 1:** Regulation of the UPR and ISR by flavivirus proteins.

Virus	Stress Pathway Activated	Viral Protein Responsible	Host Response	Effect on Virus	References
JEV	UPR	-	Interaction with BiP	Supports viral production	[43]
JEV	UPR	-	Activation of IRE-1	Supports viral replication	[36]
JEV	ISR	NS2A	Interaction with PKR, resulting in suppression	-	[60]
JEV	UPR/ISR	NS4B	Interaction with PERK, resulting in activation of PERK/ATF4/CHOP	-	[61]
JEV	ISR	-	Increases ATF3 expression	Supports viral replication	[62]
WNV	UPR	NS1-NS5	Activation of the UPR and PERK	Restricts viral replication	[39]
WNV	UPR	NS4A and NS4B	Selective activation of ATF6 and IRE-1 branches of UPR	Supports viral replication	[38]
USUV	ISR	-	Inhibition of ISR and stress granule formation	-	[63]
DENV	UPR	prM, E, NS1, NS2A, NS2B-NS3, NS4B	Activation of IRE-1	Supports viral replication	[36]
DENV	UPR	E	Interaction with calnexin, calreticulin, and BiP	Promotes viral production	[42]
DENV	UPR	-	Activation of PERK, ATF6, and IRE-1 branches	Promotes viral protein synthesis	[35,37,46,64]
DENV	UPR/ISR	NS4A	Activation of PERK	Supports viral replication	[65,66]
DENV	ISR	3′ stem loop	Interaction with G3BP1, Caprin1, TIA-1, and TIAR	Supports viral replication	[67]
DENV	UPR	-	Increased ER stress response and apoptosis	-	[68]
DENV	ISR	-	Dampened apoptotic response	Supports viral replication	[69]
ZIKV	UPR	-	Activation of IRE-1	Supports viral replication	[48,49]
ZIKV	UPR	-	Activation of PERK, ATF6, and IRE-1 branches	-	[40,41,50]
ZIKV	UPR		Activation of IRE-1/XBP1 controls immune signaling in ZIKV-infected conventional dendritic cells		[70]
ZIKV	ISR	NS3/NS4A	Activation of the ISR	-	[71]
ZIKV	ISR	C, NS3/NS2B, NS4A	Prevention of stress granule formation	-	[71]
ZIKV	ISR	Viral RNA	Interaction with G3BP1, Caprin1, TIA-1, and TIAR	Supports viral replication	[71,72,73]
ZIKV	ISR	sfRNA	Activation of PKR	Supports viral replication	[74]
ZIKV	ISR	-	Elevated expression of ATF3	Restricts viral replication	[75]
ZIKV	ISR	-	Elevated oxidative stress	-	[76]
ZIKV	ISR	-	Dampened apoptotic response	Supports viral replication	[69]
ZIKV	ISR	-	Elevated apoptotic response	-	[77]
ZIKV	ISR	C and NS5	Increased cell survival	-	[78]
YFV	ISR	-	Elevated oxidative stress	-	[79]
TBEV	UPR	-	Activation of IRE-1	Increases TBEV replication	[56]
TBEV	ISR	-	Activation of the ISR	-	[80,81]
TBEV	ISR	Viral RNA	Interaction with TIA-1 and TIAR	Restricts viral replication	[80,81]
LGTV	UPR	-	Downregulates IRE-1 and ATF6	-	[56]
LGTV	UPR/ISR	-	PERK activation	Initiation of antiviral effects	[56]

In summary, all flaviviruses examined to date activate the UPR, although the specific sensors targeted may differ (Table 1). Undoubtedly, UPR activation is important for flavivirus infection, yet the transcriptional and translational consequences of signaling through individual UPR sensors are not well understood. Characterizing these outcomes will advance our understanding of stress pathway modulation and inform the identification of therapeutic targets [17,34,44].

### 2.3. The Integrated Stress Response (ISR) Pathway

The cell is extremely sensitive to a range of internal and external stressors such as viral infection, ER stress, amino acid depletion, DNA damage and hypoxia. These insults cause homeostatic imbalances within the cell and activate an adaptive pathway referred to as the integrated stress response (ISR) [82,83]. Activation of the ISR ultimately leads to cellular responses including autophagy, apoptosis, assembly of stress granules and the innate immune response, all aimed at reinstating cellular balance [84].

The ISR is orchestrated by four different stress-inducible kinases: PERK (the ER sensor that also acts within the UPR pathway), protein kinase R (PKR), general control non-depressible 2 (GCN2) and heme-regulated inhibitor (HRI) (Figure 2) [85]. Despite the structural similarities in the kinase domains, each kinase has a distinct regulatory region, ensuring that activation of each protein is finely tuned to specific stress signals [86]. Following exposure to stress, these kinases undergo dimerization, autophosphorylation, and activation of the kinase domains. This leads to the phosphorylation of the alpha subunit of the eukaryotic translation initiation factor 2 (eIF2α) (Figure 2) [87]. Phosphorylation inactivates eIF2α by blocking the guanine nucleotide exchange factor eIF2B, thereby preventing the conversion of eIF2-GDP to eIF2-GTP. As a result, the formation of the eIF2–GTP–methionyl initiator tRNA ternary complex required for translation initiation is inhibited and protein synthesis is curtailed [88,89]. In response to flavivirus infections, for example, this blockade effectively halts cap-dependent global translation, allowing the ER time to recover from an overload of unfolded or misfolded proteins, inhibits the synthesis of viral proteins during infections, and conserves amino acids under conditions of amino acid deprivation [85,90].

While canonical cap-dependent translation is stalled, certain mRNAs such as *ATF4*, *ATF5*, and *CHOP*, critical for resolving stress by promoting cell survival or initiating cell death in response to severe or prolonged stress, are selectively translated [85,90]. The selective expression of these stress response genes likely directed by noncanonical translation mechanisms is not fully understood. One such mechanism, involves upstream open reading frames (uORF). For example, translation of *ATF4*, *ATF5* and *CHOP* is proposed to be regulated by an uORF-mediated mechanism [91,92,93]. Of these, the uORF of *ATF4* is the best studied and has a pivotal role in the ISR [94,95]. Under non-stress conditions, ATF4 protein levels are repressed due to the translation initiation from a short inhibitory uORF that precedes the downstream ATF4 coding region [94,95]. Stress conditions, however, enable the scanning pre-initiation complex to bypass the inhibitory uORF, leading to the translation of the correct ATF4-coding region [94,95].

ATF4 activates a network of downstream targets that tailor the transcriptional program for stress resolution. One downstream target is Activating Transcription Factor 3 (ATF3), a related basic leucine zipper (bZip) protein that controls the direct or indirect expression of genes vital for metabolism, apoptosis, immune response, and various cellular processes [96]. Both ATF3 and ATF4 are required for activating growth arrest and DNA damage-inducible protein 34 (GADD34) [96]. GADD34 has a crucial role in the ISR by functioning as a negative feedback regulator. Specifically, GADD34 partners with protein phosphatase 1 (PP1) to dephosphorylate eIF2α, thereby lifting translational arrest once cellular stability is restored (Figure 2) [97,98].

#### 2.3.1. Interaction Between Flaviviruses and the ISR

Flavivirus infection primarily activates the ISR through the eIF2α kinases PERK and PKR (Table 1) [84]. ISR activation suppresses global translation, promotes stress granule formation, and can result in apoptosis as part of an antiviral defense program [84]. However, flaviviruses can subvert the ISR to support replication by promoting autophagy, hijacking stress granule components, repressing ISR effectors, and enabling noncanonical translation that limits host antiviral protein synthesis [61,65,84,99]. Together, these observations underscore the context-dependent and highly adaptable role of the ISR during viral infection.

#### 2.3.2. PERK and PKR Signaling During Flavivirus Infection

In flavivirus infections, the ISR is mainly activated by the PERK and PKR kinases (Table 1). Notably, studies on DENV4 and ZIKV pinpoint PKR as the main kinase responsible for eIF2α phosphorylation during infection in human lung A549 cells [99]. Both DENV4 and ZIKV leverage PKR-mediated ISR signaling to enhance viral protein synthesis despite the translational suppression imposed by phosphorylated eIF2α [99]. This therefore benefits the virus as host proteins, including antiviral factors, are suppressed while viral proteins are continuously produced through noncanonical mechanisms, namely a putative IRES in the 5′ UTR of the viral RNA [99,100,101].

Further investigations with DENV revealed that ER stress signaling, triggered via the PERK pathway in infected human hepatoma Huh7 cells, was initiated by the NS4A protein [65,66]. This activation not only induced autophagy, as indicated by GFP-LC3 foci formation and increased LC3-II levels, but also paradoxically protected the cells from death, facilitating viral replication in epithelial and fibroblast lines [65,66,102]. DENV additionally impacted downstream ISR responses, notably interfering with stress granule (SG) assembly by sequestering essential stress granule elements such as G3BP1, Caprin-1, TIA-1 and TIAR through direct interactions with the 3′ terminal stem loop structure (3′ SL) in the viral RNA [67], thereby supporting viral replication.

ZIKV infection similarly initiates the ISR mainly driven by ZIKV proteins NS3 and NS4A [71]. Like DENV, ZIKV also blocks SG formation, a process that involves the capsid, NS3/NS2B-3 and NS4A proteins [71]. Interestingly, certain stress granule proteins such as HuR have been identified to protect against the virus [72]. ZIKV also subverts stress granule components like G3BP1, TIA-1, TIAR, and Caprin-1 via stable interactions with the viral RNA to promote efficient viral replication in Huh7, A549, human osteosarcoma-derived U2OS and human fetal astrocytes [71,72,73].

JEV infection shares similarities with DENV4 and ZIKV, with PKR and eIF2α phosphorylation occurring in later stages within mammalian A549 and 293T cells. However, JEV uniquely targets and suppresses PKR through the NS2A protein [60]. JEV infection also induces the PERK-ATF4-CHOP pathway, leading to cell death, both in vivo and in vitro, and this process is enhanced by direct interaction between NS4B and PERK [61]. Additionally, PERK activation acts as a countermeasure against JEV replication and JEV-induced apoptosis by promoting the formation of stress granules [61]. In mammalian cells, including mouse neuroblastoma cells (Neuro2a), porcine kidney stable cells (PS), human embryonic kidney cells (HEK293), and human cervical epithelial cells (HeLa), JEV has been shown to activate the stress response gene ATF3, which inhibits the autophagy pathway, thus favoring viral replication by suppressing antiviral autophagy mechanisms [62].

During WNV infection, activation of the ISR through PERK signaling induced CHOP expression and promoted apoptosis, a process linked to restriction of viral replication [39]. This antiviral effect of PERK may explain why, during infections with WNV Kunjin, ER stress response appeared to shift towards the ATF6 and IRE-1 pathways rather than PERK activation. Consistent with this idea, enhanced PERK signaling in the context of WNV Kunjin infection was shown to restrict viral replication and release [34]. Interestingly, in some WNV infections, the virus neither activated PERK nor inhibited stress granule formation [103,104]. In contrast, studies with lineage 1 and 2 chimeric WNV demonstrated that these viruses could activate PKR in response to double-stranded RNA (dsRNA) replication intermediates, causing eIF2α phosphorylation and subsequent stress granule formation, thereby supporting robust viral replication [103]. In line with this, Gilfoy and Mason reported that PKR activation in mouse embryonic fibroblast cells (MEFs) and various human cell lines infected with WNV-derived virus-like particles (VLPs) triggered PKR and led to interferon (IFN) production. These VLPs mimicked viral genome replication but were incapable of producing progeny virions, highlighting a critical role for PKR in WNV-induced interferon responses [105]. Furthermore, PRK signaling proved essential for interferon-mediated host defense in vivo, as PKR-deficient mice exhibited higher viremia levels compared to wild-type controls [105].

TBEV, like other flavivirus-infections, results in eIF2α phosphorylation, although the specific kinase responsible is yet to be determined [80,81]. TBEV prompts the formation of stress granules likely through PKR in U2OS cells and recruits stress granule -associated proteins like TIA-1 and TIAR to sites of replication through direct interactions with the viral RNA [81]. Notably, TIA-1 and TIAR are implicated in inhibiting viral translation and replication upon binding the viral RNA, underscoring an antiviral role for these proteins following the activation of the ISR [81]. Together, these studies highlight PERK- and PKR-mediated ISR signaling as central and frequently targeted components of flavivirus–host interactions (Table 1).

#### 2.3.3. HRI and GCN2 in the Context of Flavivirus Infections

In contrast to the many studies investigating the role of PERK and PKR on flavivirus infection, the significance and impact of HRI and GCN2 kinase activation within the ISR pathway is notably understudied. HRI activation helps mitigate oxidative stress [83]. In the context of WNV and DENV infection, treatment with oxidative stress inducer sodium arsenite initially triggered HRI signaling and promoted the formation of stress granules; however, both viruses gradually developed resistance to stress granule formation as the infection progressed [103,104]. Similarly, USUV infection studies demonstrated that viral replication within the ER activated stress-related cellular pathways [63]. Remarkably, USUV bypassed this defense mechanism by inhibiting eIF2α phosphorylation and stress granule assembly even in the presence of sodium arsenite [63]. Additionally ZIKV and JEV infections resulted in mitochondrial damage and the induction of oxidative stress [76,106,107,108,109]. Notably, these viruses took advantage of the oxidative stress generated during infection to temporally regulate capping of the RNA genome and replication. Consequently, antioxidant treatment during flavivirus infections was shown to reduce virus production and viral positive-to-negative strand RNA ratio, resulting in the accumulation of uncapped positive-sense viral RNAs [110]. Additionally, in vitro experiments showed that treating the NS5 RNA capping enzyme with oxidizing agents increased guanylyl transferase activity, indicating that an oxidizing environment and the resulting HRI-mediated ISR activation could increase the number of capped genomes to support more translation and replication [110].

Flavivirus-induced oxidative stress can differentially shape viral pathogenesis through inflammatory signaling pathways [111,112]. For example, in DENV infections, induction of NADPH oxidase-dependent stress activates critical immune regulators, including interferon regulatory factors 3 (IRF-3) and 7 (IRF-7), signal transducer and activator of transcription 1 (STAT-1), and nuclear factor kappa-light-chain-enhancer of activated B cells (NF-κB), causing significant damage in infected dendritic cells [113]. Associations between oxidative damage and disease severity in DENV-infected patients highlight the contribution of oxidative stress to viral pathogenesis [114,115]. Additionally, UV-inactivated JEV has been shown to enhance the levels of free radicals during infection, contributing to oxidative stress and ensuing cell death in JEV-affected mouse N8 neuronal cells [109]. Last, Muccilli and colleagues recently showed that the effective activation of the immune response to the YFV-17D vaccine strain was in part the result of the promotion of the electron transport chain and ATP synthesis, and subsequent production of reactive oxygen species, which blocked the oligomerization of the MAV receptors and initiation of interferon (IFN) [79]. Together, these studies show that several flaviviruses can induce oxidative stress, which may serve as a secondary signal to enhance the antiviral response.

In addition to inducing oxidative stress, flavivirus infection also imposes nutritional stress, particularly through depletion of amino acids as viral protein synthesis consumes host resources [96]. This nutrient deprivation suppresses global translation and promotes autophagy [82,116], resulting in the accumulation of uncharged tRNAs that activate the GCN2 kinase [117,118]. Through phosphorylation of eIF2α, GCN2 coordinates adaptive responses to nutrient stress during infection, although its role in flavivirus biology remains poorly characterized [116,118]. While the impact of GCN2 signaling on flavivirus infections has not been extensively studied, emerging evidence suggests that GCN2 can function as a host restriction factor during infection. In DENV infection, GCN2 activation limits viral replication by suppressing the NF-κB-cyclooxygenase-2 (COX-2/PGE2) pathway, which normally modulates prostaglandin E2 (PGE2) synthesis and dampens the host immune system [118,119,120,121]. GCN2 deficiency correlated with increased COX-2/PGE2 activation, whereas GCN2 overexpression produced an opposite effect in MEFs during DENV infection. Cells lacking GCN2 were also more susceptible to infection by all DENV serotypes and showed higher accumulation of DENV double-stranded RNA (dsRNA), highlighting a protective role for GCN2 in restricting viral replication [120]. Consistent with these findings, treating Huh7 cells infected with both DENV and ZIKV with halofuginone, a drug that induces the accumulation of uncharged prolyl tRNAs and activates the amino acid response pathway [122], led to a significant reduction in viral protein levels, particularly that of NS1 [123]. Treatment with halofuginone further decreased the release of viral progeny in a dose-dependent manner, thereby limiting the efficient cell-to-cell virus spread [123].

These studies collectively demonstrate how flaviviruses exploit cellular stress responses, including oxidative stress and amino acid deficiencies, to enhance their virulence and evade host defenses. To date, the downstream effectors and transcriptional programs induced by HRI and GCN2 in response to stress such as flavivirus infection have not been investigated. Understanding the specific viral proteins involved in the activation of the respective kinases and the downstream effects could provide important links to autophagy and immune modulation, two pathways with significant effects on flavivirus infection. Such investigations would also offer promising avenues for novel therapeutic interventions.

Collectively, these studies identify the ISR as a central and adaptable host pathway during flavivirus infection. PERK and PKR represent the dominant ISR sensors, whereas HRI and GCN2 appear to modulate infection in response to oxidative and nutritional stress in a context-dependent manner. How flaviviruses differentially engage or evade these ISR branches to shape translation, replication, and immunity remains an important area for future investigation.

## 3. Balancing Stress, Immunity and Cell Fate During Flavivirus Infection

Flavivirus infection elicits a coordinated cellular stress response encompassing the UPR, ISR, innate immunity, apoptosis, and autophagy. Once viewed as independent processes, these pathways are now recognized to interact during infection, as discussed in the following sections.

### 3.1. Crosstalk Between UPR, ISR and Innate Immunity Pathways During Flavivirus Infections

The UPR and ISR are closely interconnected, most notably through shared PERK signaling [17,61], and together shape cellular stress adaptation and innate immune responses. Increasing evidence indicates that UPR signaling intersects with antiviral sensing pathways during flavivirus infection. In DENV, ZIKV, WNV, and TBEV infections, UPR activation precedes IRF3-dependent interferon signaling, suggesting a role in early antiviral priming that enhances innate immune activation and restricts viral replication [55]. Consistent with this, co-stimulation studies demonstrate synergistic interactions between UPR signaling and innate immune pathways, whereby activation of the IRE-1/XBP1 axis or induction of ER stress enhances type I interferon responses to Toll-like receptor (TLR) ligands and the dsRNA mimic poly(I:C) in macrophages and dendritic cells [124,125,126].

Beyond its role in interferon signaling, each UPR branch has been increasingly implicated in the regulation of proinflammatory responses. IRE-1α engages TRAF2 to promote NF-κB activation, while ATF6 and PERK signaling also converge on NF-κB through AKT activation and translational repression of its inhibitor IκB, respectively [127]. PERK further contributes to inflammation via ATF4-dependent induction of IL-6, synergizing with TLR signaling pathways [128]. In addition, all three UPR arms activate MAPK pathways, including JNK, p38, and ERK1/2, thereby shaping cytokine production [129]. Reciprocal regulation has also been described, whereby antiviral signaling through pattern recognition receptors enhances UPR activation; notably, TLR2 and TLR4 signaling promotes IRE-1α/XBP1 activation via TRAF6 and NOX2, supporting sustained proinflammatory cytokine production in macrophages [130,131].

During flavivirus infection, the viral RNA genomes are degraded by the cellular 5′-to-3′ exonuclease Xrn1 [132]. Notably, the 3′ UTR of flaviviral genomes harbor single or tandem pseudoknot structures that stall Xrn1 degradation resulting in the accumulation of small flaviviral RNAs (sfRNAs) [133,134]. These sfRNAs, through RNA–protein interactions, are proposed to sequester and dampen cellular innate immune responses [135,136,137,138,139,140]. Pallarés and colleagues showed that ZIKV sfRNAs activate PKR, which, through phosphorylation of eIF2α, limits global translation, including that of type I interferons and interferon-stimulated factors [74]. Thus, activation of PKR and induction of the ISR serves to promote replication and the production of new virions [74]. Further, mediators of the UPR, such as IRE-1α, are targeted to influence the antiviral response [49,70]. However, the cell acts to balance this manipulation of the immune response. Through inhibitor and RNA interference approaches, ZIKV infection was found to activate ATF3, a stress response gene, via the ISR-ATF4 signaling pathway [75]. Interestingly, depletion of ATF3 led to increased viral gene expression and virion production, indicating a restrictive role for ATF3 achieved through modulation of antiviral interferon response [75]. Notably, this study contrasted an earlier study with JEV that reported ATF3 interacting with select promoter sites and the inhibition of expression of innate immune response signaling factors *STAT1*, *IRF9* and *ISG15* to support JEV infection [62]. These studies illustrate that activation of the same stress response pathways by different flaviviruses can yield opposing effects on innate immune signaling, either restricting or promoting antiviral responses.

Although the role of the UPR and ISR in priming antiviral responses during flavivirus infection has begun to emerge [55], how UPR-ISR signaling coordinates interferon and inflammatory outputs remains poorly defined. Key unresolved questions include whether UPR- and ISR-specific factors directly regulate interferon or cytokine gene expression and whether such mechanisms are conserved across flaviviruses. Addressing these gaps will be critical for understanding how cellular stress responses interface with antiviral immunity.

### 3.2. UPR and ISR Signaling in the Regulation of Apoptosis During Flavivirus Infection

Apoptosis is a host defense program that eliminates damaged or pathogen-infected cells and strongly influences viral infection outcomes [141]. Coordinated signaling between the ER and mitochondria positions apoptosis as an integrative node for cellular stress responses [142].

UPR-induced apoptosis can be initiated through multiple signaling branches. Activation of IRE-1α promotes recruitment of TRAF2 and ASK1, triggering downstream kinase cascades that induce pro-apoptotic factors such as Bax, Bak, Bim, PUMA, and NOXA, to ultimately engage mitochondrial apoptosis [143]. ATF6 has also been implicated in apoptotic regulation through mechanisms including induction of DAPK1 and CHOP expression [144,145]. Similarly, PERK-eIF2α-ATF4 signaling drives apoptosis via CHOP-dependent transcriptional programs that enhance pro-apoptotic gene expression while repressing anti-apoptotic factors [39,52]. Collectively, studies in DENV, JEV, WNV, and ZIKV infections highlight extensive interplay between UPR signaling and apoptotic pathways.

In DENV infections, persistent UPR signaling triggers a switch from pro-survival to pro-apoptotic states, promoting apoptotic cell death in host cells. In HepG2 cells infected with DENV2, there was a notable increase in spliced *XBP1* mRNA and BiP protein levels, and a reduced association of BiP with PERK or ATF6 receptors, indicating an activated ER stress response (Figure 1) [68]. This activation resulted in elevated expression of apoptosis-related genes like *Noxa*, *Puma*, and *CHOP*, along with cleavage of caspases-4, -7, -8, and -9, suggesting induction of apoptosis by DENV-induced UPR [68]. Furthermore, interaction between various ER stress pathways and cell death mechanisms have been observed in human monocytic (U937) cells infected with DENV [146]. DENV1 infection in Huh7 cells was also shown to trigger ER stress and apoptosis pathways, primarily through the accumulation of viral proteins capsid and prM at the ER, which induced downregulation of mitochondrial membrane potential and expression of p53 [147]. In contrast, infection of human fibrosarcoma 2fTGH cells with the PL046 strain of DENV2 activated the IRE-1/XBP1 and ATF6 arms of the UPR to induce CHOP and GADD34 expression [35]. Notably, CHOP expression did not affect Bcl2, procaspase-9, procaspase-3 and PARP protein levels, consistent with the absence of apoptotic signaling [35].

JEV strongly induced ER stress and UPR by upregulating the expression of ER chaperones in BHK-21 cells. This induction of the UPR subsequently led to increased CHOP expression and facilitated caspase-mediated apoptosis [45]. Activation of ER sensor PERK by JEV in neuronal cells and mouse brains similarly initiated downstream apoptotic processes, further highlighting the role of the UPR and ISR pathways in apoptosis and encephalitis induced by JEV, including through the NS4B viral protein [61].

WNV infection of the central nervous system in patients leads to neuronal cell death by apoptosis [148,149,150,151]. This pathology is mirrored in animals and embryonic stem cell-derived neurons [152,153], with caspase-3 directing apoptosis in the brains of wild-type mice [152]. WNV infection in SK-N-MC neuroblastoma cells activates the IRE-1/XBP1 and PERK UPR pathways but induces the degradation of ATF6 [39]. The extended PERK-ISR signaling induced ATF4-directed expression of CHOP in both SK-N-MC cells and primary rat hippocampal neuronal/glial cultures and subsequent caspase-3 activation and proteolytic cleavage of PARP, both indicators of apoptosis [39]. Expression of WNV nonstructural proteins were shown to impact CHOP expression [39]. Conversely, MEFs infected with the WNV Kunjin initiated IRE-1 and ATF6 UPR pathways but blocked PERK activation [53]. This adaptation allowed for the virus to utilize ATF6 and stymie the PERK pathway, thereby limiting CHOP expression and downstream apoptosis, which ultimately increased viral levels [53]. WNV infections have also been implicated in apoptosis of midgut cells of infected *Culex pipiens pipiens* mosquitoes; however, the role of ER stress in activating apoptosis remains to be determined [154].

ZIKV triggers apoptosis in placental trophoblasts through the activation of IRE-1/XBP1 and CHOP [144]. This is complemented by the activation of JNK and MAPK signaling effector proteins such that blocking JNK significantly reduces ZIKV-induced apoptosis in these cells [144]. Further, apoptosis in response to ZIKV infection is observed in vivo in the hippocampus and cortex regions of the brain in adult mice [77]. Other studies likewise established the UPR as a central mechanism driving apoptosis in ZIKV-infected human and mouse neurons and neural cells [50,51]. Interestingly, in certain instances, the ZIKV-induced ER stress response might be restricted, as the virus suppresses the expression of CHOP proteins despite the presence of the mRNA transcript [144]. This mechanism likely serves to inhibit cell death, thereby promoting viral replication [52]. Moreover, ZIKV may take steps to silence infection-induced apoptosis by targeting downstream mediators of the response. A recent study demonstrated the ZIKV-encoded capsid and NS5 viral proteins mediating the inhibition of host NLRP3 and A20 post-transcription, diminishing apoptosis and promoting cell survival by overactivation of the NF-κB pathway [78]. Additionally, complexes tethering the ER to the mitochondria have been established as targets of ZIKV and DENV. In particular, the targeting of the RRBP1-SYNJ2BP tethering complex by ZIKV results in a dampened apoptotic response and likely serves to support viral replication [69].

Taken together, these findings highlight the UPR as a key determinant of apoptotic outcomes during flavivirus infection, with effects that depend on viral and cellular context. While sustained UPR/ISR signaling can promote CHOP-dependent apoptosis, selective engagement or suppression of specific signaling branches may instead favor cell survival and viral replication, underscoring the complex role of stress signaling in shaping disease outcomes.

### 3.3. Stress Signaling and Autophagic Outcomes During Flavivirus Infection

Autophagy is a central stress-adaptive pathway that functions in concert with the UPR and ISR to maintain cellular homeostasis during virus-induced stress. Through lysosome-dependent degradation of damaged organelles and proteins, autophagy promotes cell survival and contributes to both innate and adaptive antiviral immunity, including interferon production and antigen presentation to T lymphocytes [155,156,157,158,159]. Autophagy is induced downstream of multiple stress signals, including ER stress, nutrient deprivation, hypoxia, and pathogen-associated molecular patterns, and is closely linked to UPR and ISR signaling [155]. All three UPR branches, as well as ISR activation through eIF2α phosphorylation, have been implicated in autophagy induction via transcriptional programs involving ATF4, CHOP, XBP1, and ATF6, which regulate core autophagy-related genes such as LC3, ATG5, and Beclin-1 [160,161,162,163,164,165,166].

Flaviviruses can subvert ER–autophagy coupling to support infection by exploiting autophagosome-derived double-membrane compartments that concentrate viral components and shield viral RNA from innate immune sensing, thereby facilitating replication [15,158]. Consistent with this, ER stress-associated autophagy has been observed during DENV, JEV, and USUV infections [54,167,168].

DENV co-opts autophagy to promote viral replication [64,167,169]. In HepG2-infected cells, LC3-II, an autophagosomal marker, co-localized with DENV dsRNA and NS1, indicating that viral replication occurred within autophagic vacuoles [64]. In vitro and in the AG129 mouse model studies using spautin-1, an autophagy inhibitor, also suggested that the virus relies on autophagy for the production of infectious viral particles, thus enhancing viral replication while evading lysosomal degradation [169]. The ER stress response activated by DENV was crucial for triggering autophagy across various mammalian cell types. Specifically, the PERK-eIF2α and IRE-1α-JNK signaling pathways were identified as promoters of autophagy, leading to increased viral load upon DENV infection [170]. However, the ATF6-related pathway did not influence autophagy or viral replication [170]. Subsequent in vivo experiments using a JNK inhibitor confirmed these findings, showing reduced viral titers, the amelioration of disease symptoms, and increased survival rates. Together, these results suggest that DENV2-induced ER stress enhanced autophagy activity, viral replication, and pathogenesis through the IRE-1 and PERK UPR signaling pathways, both in vitro and in vivo [170].

Similarly, in the case of JEV, ER stress and the UPR played a significant role in triggering autophagy, which negatively regulated infection in different cell lines. This was demonstrated in neuronal cells, where blocking the PERK-eIF2α pathway through siRNA-mediated depletion and a PERK inhibitor did not affect autophagy or JEV replication [168]. However, XBP1 and ATF6 were shown to modulate autophagy either individually or together, as depletion of the proteins inhibited the activation of autophagy and notably increased JEV-induced cell death [168]. Additionally, the role of oxidative stress in JEV-induced autophagy has also been investigated, with recent studies showing that diphenyleneiodonium, an antioxidant chemical, prevented JEV-induced autophagy by blocking the UPR pathway, hence reducing JEV production in neuronal Neuro2a cells [171]. ATF3, a stress-induced transcription factor that is activated by the ISR ATF4 master regulator, was reported to be a negative regulator of autophagy in JEV-infected cells [62]. Specifically, in ATF3 RNAi-depleted cells, JEV expression increased coincident with increased levels of *ATG* genes. Moreover, chromatin immunoprecipitation of ATF3 in these cells showed ATF3 binding the *ATG5* promoter region [62]. USUV, another virus in the JEV serocomplex, was also shown to concurrently activate the UPR, particularly the IRE-1/XBP1 branch, along with autophagy during infection. This suggests a potential involvement of autophagy during USUV infection [54]. While additional research is needed to clarify the underlying mechanism of the interaction between the UPR and activation of autophagy, this study indicates concomitant outcomes of these pathways.

While there are reports of autophagy during ZIKV and WNV infection, unlike DENV and JEV, no studies have directly linked activation of the UPR or the ISR to autophagy induction during these infections, despite both viruses being known to activate these signaling cascades. Nevertheless, an indirect connection exists, such as through flavivirus-induced ER membrane rearrangements, which contribute to ER stress. ZIKV infection increased in human fetal neural stem cells following the activation of autophagy [172]. Expression of ZIKV NS4A and NS4B also triggered distinct autophagy markers [172] by inhibiting Akt-mTOR signaling, a key pathway regulating cell growth, differentiation and survival [173]. While the discussion above has focused on nonselective macroautophagy, it is worth noting that ZIKV interfaces with mitophagy, a selective macroautophagy pathway that removes dysfunctional mitochondria, to safeguard mitochondria homeostasis and function [174]. Given that the mitochondrial antiviral (MAV) signaling protein is embedded in the outer mitochondrial membrane, maintaining the integrity of the organelle is central to regulating innate immune signaling [174]. Ponia and colleagues report that ZIKV NS5 blocked the activation of the mitophagy pathway [175]. The inability to remove damaged mitochondria, however, tipped the balance towards the induction of the ISR. Specifically, exposed mitochondrial RNAs activated PKR to promote the expression of proinflammatory chemokines, which counterintuitively increases the tissue distribution of ZIKV [175]. WNV infection also induced autophagy [176]; however, depletion of key autophagy-related genes *ATG5* and *ATG7* did not hinder WNV replication or infectious particles and suggested that WNV-induced autophagy was not required for the WNV infectious cycle [176,177,178].

Together, these studies indicate that autophagy is commonly engaged during flavivirus infection but is differentially exploited in a virus- and context-dependent manner. The variable coupling of autophagy to UPR–ISR signaling underscores important gaps in our understanding of how stress pathways shape viral replication and disease outcomes.

## 4. Conclusions

Flavivirus infections activate the UPR and ISR through ER stress, metabolic and oxidative perturbations, and viral dsRNA, engaging adaptive programs that shape cellular survival and infection outcomes. These pathways intersect with antiviral immunity and autophagy, yet prolonged stress can shift signaling toward cell death. Although UPR and ISR sensor engagement during flavivirus infection is increasingly well defined, the downstream transcriptional and translational consequences and how flaviviruses subvert these effectors remain poorly understood. Addressing these gaps through integrated molecular and omics-based approaches will be essential for advancing our understanding of flavivirus pathogenesis and identifying new antiviral targets.

## Data Availability

No new data were created or analyzed in this study.

## Abbreviations

The following abbreviations are used in this manuscript:

ASK1	Apoptosis signal-regulating kinase 1
ATF3	Activating Transcription Factor 3
ATF4	Activating Transcription Factor 4
ATF6	Activating transcription factor 6
BiP	Binding Immunoglobulin Protein
bZIP	Basic leucine zipper
C	Capsid protein
DAPK	Death-associated protein kinase 1
DENV	Dengue virus
dsRNA	Double-stranded RNA
E	Envelope protein
ER	Endoplasmic reticulum
ERAD	ER-associated protein degradation
eIF2	Eukaryotic translation initiation factor 2
GADD34	Growth arrest and DNA damage-inducible protein 34
GCN2	General control non-depressible 2
HRI	Heme-regulated inhibitor
IFN	Interferon
IRE-1	Inositol requiring enzyme 1
IRF	Interferon regulatory factors
ISR	Integrated stress response
JEV	Japanese encephalitis virus
JNK	c-Jun amino-terminal kinase
LGTV	Langat virus
MAPK	Mitogen-activated protein kinases
MAV	Mitochondrial antiviral signaling protein
MEFs	Mouse embryonic fibroblast cells
NF-κB	Nuclear factor kappa-light-chain-enhancer of activated B cells
PERK	PKR-like ER kinase
PKR	Protein kinase R
prM/M	Pre-membrane/membrane protein
RIDD	Regulated IRE-1 dependent decay
sfRNA	Small flaviviral RNA
SG	Stress granules
STAT-1	Signal transducer and activator of transcription 1
TBEV	Tick-borne encephalitis virus
TMUV	Tembusu virus
TLR	Toll-like receptor
TRAF2	TNF receptor-associated factor 2
UPR	Unfolded protein response
uORF	Upstream open reading frame
UTR	Untranslated region
USUV	Usutu virus
VLPs	Virus-like particles
WNV	West Nile virus
YFV	Yellow fever virus
ZIKV	Zika virus
